# Pirfenidone exacerbates Th2-driven vasculopathy in a mouse model of systemic sclerosis-associated interstitial lung disease

**DOI:** 10.1183/13993003.02347-2021

**Published:** 2022-10-20

**Authors:** Anna Birnhuber, Katharina Jandl, Valentina Biasin, Elisabeth Fließer, Francesco Valzano, Leigh M. Marsh, Christina Krolczik, Andrea Olschewski, Jochen Wilhelm, Wolfgang Toller, Akos Heinemann, Horst Olschewski, Malgorzata Wygrecka, Grazyna Kwapiszewska

**Affiliations:** 1 Ludwig Boltzmann Institute for Lung Vascular Research Graz, Graz, Austria; 2 Otto Loewi Research Center, Division of Physiology, Medical University of Graz, Graz, Austria; 3 Otto Loewi Research Center, Division of Pharmacology, Medical University of Graz, Graz, Austria; 4 Center for Infection and Genomics of the Lung, Universities of Giessen and Marburg Lung Center, Member of the German Center for Lung Research, Giessen, Germany; 5 Dept of Anaesthesiology and Intensive Care Medicine, Medical University of Graz, Graz, Austria; 6 Dept of Internal Medicine, Universities of Giessen and Marburg Lung Center, Giessen, Germany; 7 Division of Pulmonology, Dept of Internal Medicine, Medical University of Graz, Graz, Austria; 8 Institute for Lung Health (ILH), Justus Liebig University, Giessen, Germany

## Abstract

**Background:**

Systemic sclerosis (SSc) is an autoimmune disease characterised by severe vasculopathy and fibrosis of various organs including the lung. Targeted treatment options for SSc-associated interstitial lung disease (SSc-ILD) are scarce. We assessed the effects of pirfenidone in a mouse model of SSc-ILD.

**Methods:**

Pulmonary function, inflammation and collagen deposition in response to pirfenidone were assessed in Fra-2-overexpressing transgenic (Fra-2 TG) and bleomycin-treated mice. In Fra-2 TG mice, lung transcriptome was analysed after pirfenidone treatment. *In vitro*, pirfenidone effects on human eosinophil and endothelial cell function were analysed using flow cytometry-based assays and electric cell-substrate impedance measurements, respectively.

**Results:**

Pirfenidone treatment attenuated pulmonary remodelling in the bleomycin model, but aggravated pulmonary inflammation, fibrosis and vascular remodelling in Fra-2 TG mice. Pirfenidone increased interleukin (IL)-4 levels and eosinophil numbers in lung tissue of Fra-2 TG mice without directly affecting eosinophil activation and migration *in vitro*. A pronounced immune response with high levels of cytokines/chemokines and disturbed endothelial integrity with low vascular endothelial (VE)-cadherin levels was observed in pirfenidone-treated Fra-2 TG mice. In contrast, eosinophil and VE-cadherin levels were unchanged in bleomycin-treated mice and not influenced by pirfenidone. *In vitro*, pirfenidone exacerbated the IL-4 induced reduction of endothelial barrier resistance, leading to higher leukocyte transmigration.

**Conclusion:**

This study shows that antifibrotic properties of pirfenidone may be overruled by unwanted interactions with pre-injured endothelium in a setting of high T-helper type 2 inflammation in a model of SSc-ILD. Careful ILD patient phenotyping may be required to exploit benefits of pirfenidone while avoiding therapy failure and additional lung damage in some patients.

## Abstract 2

**Pirfenidone worsens vascular and pulmonary dysfunction in a SSc-ILD mouse model due to its negative effects on primed endothelial cells and may lead to unfavourable effects in patients with underlying type 2 inflammation as seen in SSc-ILD** https://bit.ly/3Jk821j

## Introduction

Systemic sclerosis (SSc) is an autoimmune disease leading to severe vasculopathy and fibrosis of various organs. It is characterised by vascular and fibrotic abnormalities, due to dysregulation of innate and adaptive immunity leading to high levels of inflammatory and pro-fibrotic mediators such as interleukin (IL)-1, IL-6, tumour necrosis factor (TNF)-α or transforming growth factor (TGF)-β, as well as the T-helper type 2 (Th2) cytokines IL-4 and IL-13 [1, 2]. A growing body of evidence highlights SSc as a vascular disease with a prominent role of endothelial dysfunction. Endothelial cells and blood vessels seem to be the initial target of injury in all organs affected by the disease [3, 4]. Lung involvement, leading to interstitial lung disease in SSc (SSc-ILD) is very frequent and accounts for the majority of SSc-associated deaths [5, 6]. The underlying pathomechanisms are not fully elucidated, therefore disease-modifying treatment options for SSc-ILD are limited and therapy is mostly confined to systemic immunosuppression [7, 8]. The tyrosine kinase inhibitor nintedanib, previously used for the treatment of idiopathic pulmonary fibrosis (IPF), was only recently approved for the treatment of rapidly progressive SSc-ILD [9].

A second antifibrotic drug, pirfenidone, has long been discussed for SSc-ILD therapy. Pirfenidone reduces pro-inflammatory and fibrotic responses by decreasing levels of inflammatory mediators and growth factors such as IL-1β or TGF-β, as shown in multiple cell culture studies, animal models and in IPF patients [10–14]. Although pirfenidone treatment does not cure IPF, it delays the decline in forced vital capacity and disease progression [12, 15]. While pirfenidone is approved as treatment for IPF, it is not approved for other forms of pulmonary fibrosis.

The RELIEF study investigated the effects of pirfenidone in patients with non-IPF progressive fibrotic ILD, but was terminated prematurely due to slow enrolment [16]. In this study, pirfenidone delayed the decline in forced vital capacity without a significant increase in adverse effects. However, due to low patient numbers, subgroup analysis for SSc-ILD was not possible [16]. Therefore, the question remains open whether SSc-ILD patients may benefit from pirfenidone treatment or whether they may be prone to adverse effects.

We tested the hypothesis that pirfenidone might be beneficial in SSc-ILD due to its anti-inflammatory and antifibrotic effects, by applying a mouse model overexpressing the AP-1 transcription factor Fos-related antigen-2 (Fra-2 transgene) [17]. This model recapitulates major features of human SSc, such as early alterations of the vasculature with endothelial cell apoptosis [18], vascular remodelling [19] and development of pulmonary hypertension (preceding the onset of fibrosis), systemic inflammation [17, 20, 21] and fibrosis of skin and other internal organs, including the lungs [17, 22, 23]. Similar to SSc patients, Fra-2 TG mice have high levels of inflammatory mediators such as IL-1β and IL-6, and of Th2 cytokines such as IL-4 and IL-13 [1, 2, 17, 20], thus representing a valuable model to test drug candidates for SSc-ILD patients.

In contrast to our expectations, pirfenidone aggravated the vascular and pulmonary phenotype in the Fra-2 mouse model of SSc-ILD. We provide evidence that in certain specific inflammatory settings, pirfenidone may have detrimental effects on endothelial cell function and lung permeability.

## Material and methods

A detailed description of all methods is provided in the supplementary material.

### Animal experiments

**FIGURE 1:**
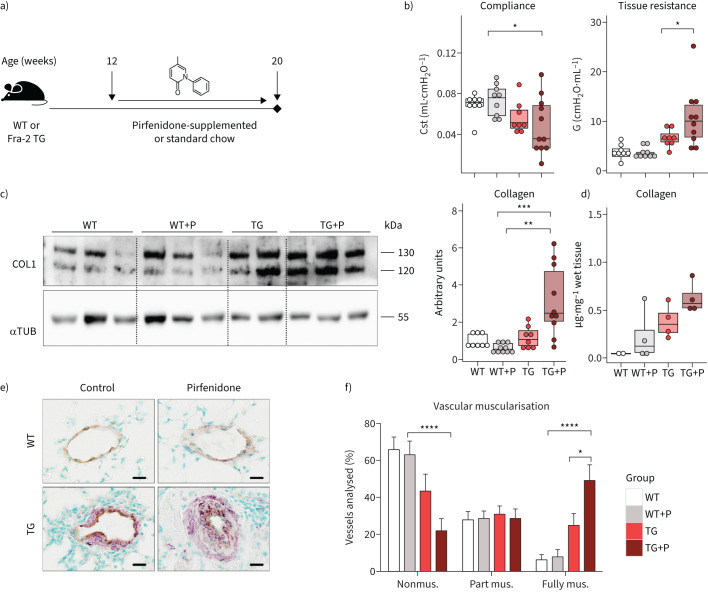
Pulmonary remodelling is worsened following pirfenidone treatment in Fra-2-overexpressing transgenic (Fra-2 TG) mice. a) Schematic representation of pirfenidone (P) treatment in Fra-2 TG and wild-type (WT) mice. Lung function measurements and organ collection was performed in ∼20-week-old mice after 8 weeks of pirfenidone treatment. b) Lung function measurements showing quasi-static compliance (Cst) and tissue dampening (G). c) Western blot analysis and corresponding quantification of Collagen I (COL1) in lung homogenates of WT and TG mice with (+P) and without pirfenidone. α-tubulin (αTUB) served as loading control. One of two Western blots is shown. d) Hydroxyproline measurement of collagen in lung tissue. Data are indicated as boxplots with dot-plot overlays. Statistical analysis was performed using nonparametric Kruskal–Wallis testing with post-analysis to compare specific groups. e) Representative images of double immunohistochemical staining for von Willebrand factor (brown) and α-smooth muscle actin (purple). Scale bars=10 µm. f) Percentage of nonmuscularised (nonmus.), partially muscularised (part mus.) and fully muscularised (fully mus.) vessels <100 μm in diameter. n=4 (TG) or n=6 (WT, WT+P and TG+P). Data are shown as mean±sd. *: p<0.05, **: p<0.01, ***: p<0.001, ****: p<0.0001.

Female Fra-2 overexpressing/transgenic (Fra-2 TG) mice and wild-type (WT) littermates were maintained under specific pathogen-free conditions in isolated ventilated cages with 12-h light/dark cycles. All animal experiments met European Union guidelines (2010/63/EU) and were approved by the local authorities (Austrian Ministry of Education, Science and Culture). Bleomycin was given intratracheally at a dose of 0.8 units per kg bodyweight, as described previously [24, 25]. Pirfenidone treatment protocol was adapted from previous publications [26] and incorporated into standard laboratory chow at 2.8 mg·g^−1^ (Sniff, Soest, Germany). Pirfenidone was provided by Hoffmann–La Roche. Food was weighed regularly and mean pirfenidone uptake was calculated. Mean pirfenidone uptake was ∼400 mg·kg^−1^ bodyweight per day, a frequently used dosage in mouse studies, leading to pirfenidone exposure ratios and plasma concentrations similar to those in patients treated with pirfenidone [10, 27, 28]. Pirfenidone treatment of Fra-2 TG and WT mice was performed in two independent experiments with five to eight mice per group. Schematic representations of Fra-2 TG and bleomycin experiments are shown in figures 1a and 5a, respectively.

## Results

### Pirfenidone aggravates pulmonary remodelling in the Fra-2 TG mouse model

11- to 12-week-old Fra-2 TG and WT mice received pirfenidone-supplemented food over the course of 8 weeks (figure 1a). As compared to mice on standard chow, pirfenidone treatment worsened lung function in Fra-2 TG mice, as indicated by decreased quasi-static compliance and significantly increased tissue damping, a parameter related to tissue resistance (figure 1b). Increased collagen deposits were observed predominantly in perivascular regions of the lungs of Fra-2 TG mice (supplementary figure S1). Collagen quantification by Western blot analysis and hydroxyproline measurements revealed significantly elevated collagen levels in the lungs of pirfenidone-treated Fra-2 TG mice as compared to WT control mice (figure 1c and d). In addition to parenchymal alterations, Fra-2 TG mice had enhanced muscularisation of small pulmonary vessels, which was aggravated upon pirfenidone treatment (figure 1e and f). This indicates a negative influence of pirfenidone on the pulmonary circulation. Of note, pirfenidone did not affect lung function, pulmonary architecture, collagen deposition or vessel muscularisation in WT mice.

### Pirfenidone treatment increases inflammatory infiltration and eosinophilia in Fra-2 TG mice

**FIGURE 2:**
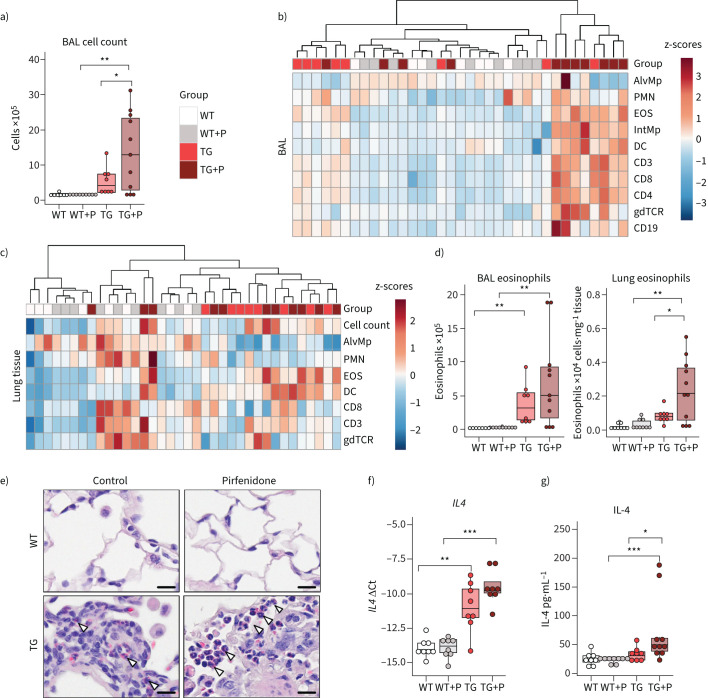
Pirfenidone increases inflammatory cell counts in the bronchoalveolar lavage (BAL) and eosinophilic infiltration into the lung tissue in Fra-2-overexpressing transgenic (Fra-2 TG) mice. a) Inflammatory cell count in the BAL of wild-type (WT) and Fra-2 TG mice with (+P) and without pirfenidone treatment. b and c) Heat map representations with hierarchical clustering of relative proportions of inflammatory cell populations in b) BAL and c) lung tissue of WT and TG mice with (+P) and without pirfenidone treatment. Data were normalised using sqrt(sqrt(cellcount)); z-scores are shown. d) Eosinophil cell count in BAL and lung tissue of WT and TG mice with (+P) and without pirfenidone treatment. e) Chromotrop 2R staining of eosinophil granules (arrowheads) in the lung tissue of WT and TG mice with and without pirfenidone treatment. Scale bars=10 µm. f) Quantitative real-time PCR analysis of interleukin-4 (*IL4*) gene expression. g) IL-4 protein content in lung tissue homogenates. Data are presented as boxplots with dot-plot overlays. AlvMp: alveolar macrophages; PMN: polymorphonuclear granulocytes/neutrophils; EOS: eosinophils; IntMp: interstitial macrophages; DC: dendritic cells; CD3: CD3^+^ T-cells; CD8: CD8^+^ cytotoxic T-cells; CD4: CD4^+^ T-helper cells; gdTCR: γδ T-cell receptor positive cells; CD19: CD19^+^ B-cells. Statistical analysis was performed using nonparametric Kruskal–Wallis testing with post-analysis to compare specific groups. *: p<0.05, **: p<0.01, ***: p<0.001.

As shown previously [17, 20, 21], Fra-2 TG mice had elevated levels of inflammatory cells in the bronchoalveolar lavage fluid (BALF) (figure 2a), which was further exacerbated by pirfenidone (figure 2a). While almost all inflammatory cell populations were increased in the BALF (figure 2b), alterations in the lung tissue were dominated by increased eosinophil levels (figure 2c–e). In addition, gene expression and protein levels of IL-4 were elevated in the lungs of Fra-2 TG mice (figure 2f and g). Pirfenidone did not alter the inflammatory profile or lung function in WT mice, indicating that pirfenidone by itself has no harmful effects on the healthy lung.

Of note, intranasal application of the glucocorticoid budesonide in Fra-2 TG mice ameliorated pulmonary inflammation, improved lung function [21] and decreased collagen deposition in the lung parenchyma (supplementary figure S2). This highlights the role of inflammation in the development of pulmonary fibrosis in this mouse model.

To investigate whether increased influx of eosinophils into the lung tissue might be due to direct effects of pirfenidone on eosinophils, we analysed stimulation-induced shape change, chemotaxis, reactive oxygen species production and survival of human eosinophils isolated from blood in response to pirfenidone. Pirfenidone alone did not affect eosinophil activation as measured by shape change (supplementary figure S3a) or reactive oxygen species production (supplementary figure S3b). Eotaxin-induced shape change and chemotaxis remained unaltered by pirfenidone pre-treatment (supplementary figure S3c and d). Survival analysis showed that pirfenidone, alone or in combination with pro-survival factor IL-5, had no influence on eosinophil longevity *in vitro* (supplementary figure S3e). In summary, pirfenidone did not alter eosinophil properties or function by its own, nor did it modulate eotaxin or IL-5 mediated effects on shape change, chemotaxis or survival. Therefore, increased eosinophil abundance in the lungs of pirfenidone-treated Fra-2 TG mice cannot be explained by direct pirfenidone effects on eosinophils.

### Transcriptomic profiling highlights inflammatory pathways upregulated upon pirfenidone treatment in Fra-2 TG mice

**FIGURE 3:**
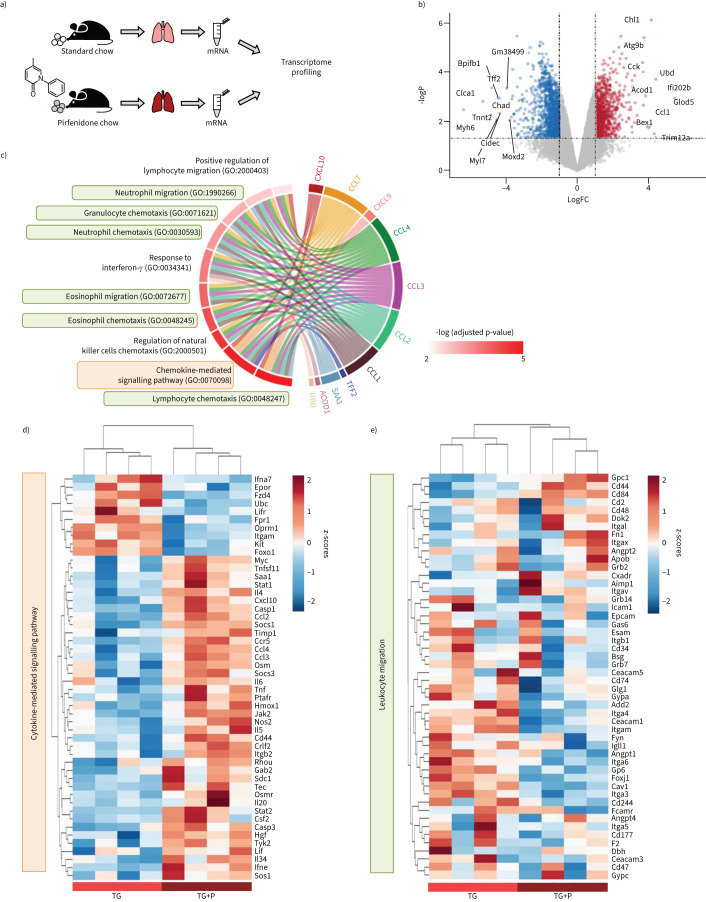
Transcriptomic profiling highlights inflammatory pathways upregulated upon pirfenidone treatment in Fra-2-overexpressing transgenic (Fra-2 TG) mice. a) Schematic representation of the experimental setup. b) Volcano plot showing differential gene regulation in Fra-2 TG mouse lungs with (+P) and without pirfenidone. Top 10 regulated genes according to their log-fold-change (logFC) are labelled by name. c) Top 10 significantly regulated gene ontologies (GO biological process) and their significantly regulated genes within the dataset. d and e) Heatmap representation with hierarchical clustering of genes within the gene ontologies d) GO:0019221 cytokine-mediated signalling pathway and e) GO:0050900 leukocyte migration.

Next, we sought to describe the molecular processes induced by pirfenidone, that may explain the increased eosinophil numbers and worsened phenotype in Fra-2 TG mice. To this end, we performed transcriptional profiling of lung tissue from Fra-2 TG mice with and without 8 weeks of pirfenidone treatment (figures 1a and 3a). The top 10 significantly up- or down-regulated genes, as defined by absolute log-fold-change (logFC) (figure 3b), highlighted main changes in the lung. Several genes associated with mucosal immunity (BPI Fold Containing Family B Member 1 (Bpifb1)) or epithelial repair (Trefoil factor 2 (Tff2)) and function (Chloride Channel Accessory 1 (Clca1)) were downregulated, possibly indicating increased epithelial damage in Fra-2 TG mice following pirfenidone treatment. Within the strongest upregulated genes, most genes were linked to innate immunity (interferon-activated gene 202B (Ifi202b), ubiquitin D (Ubd), tripartite motif containing 12A (Trim12a), aconitate decarboxylase 1 (Acod1), CC-chemokine ligand 1 (Ccl1)), to eosinophils (glyoxalase domain containing 5 (Glod5)) and to inflammatory diseases (cell adhesion molecule L1 like (Chl1), Acod1) (figure 3b). Two of the top 10 upregulated genes were also connected to an IPF-specific gene signature (cholecystokinin (Cck) and Chl1) [29]. These data point towards a dysregulation of the immune response and concomitant lung injury upon pirfenidone treatment in Fra-2 mice.

Gene enrichment analysis showed significant overrepresentation of altered gene expression in inflammatory pathways, such as chemotaxis and migration of lymphocytes, eosinophils and neutrophils, and the regulation thereof (figure 3c). The top 10 most significant gene ontologies were marked by the increased expression of chemokines such as Ccl1, Ccl2, Ccl3, Ccl4, Ccl7, Cxcl9 and Cxcl10 (figure 3c). Unbiased hierarchical clustering of all genes of the two main parent gene ontologies, namely the cytokine-mediated signalling pathway (GO:0019221: orange background, figure 3d) and leukocyte migration (GO:0050900: green background, figure 3e), showed clear separation of Fra-2 TG lung homogenates of mice with pirfenidone treatment compared to Fra-2 TG mice without pirfenidone treatment. In addition to upregulation of inflammatory cytokines such as Il-4, Ccl2/3/4 and transcription factors, such as Stat1/2 (figure 3d and e), numerous endothelial cell junction or cell-contact proteins such as Ceacam1 (Carcinoembryonic Antigen-Related Cell Adhesion Molecule 1), Add2 (Adducin 2) or integrins (Itga3, Itga4) were downregulated (figure 3e).

### Pirfenidone leads to aggravated loss of vascular endothelial cadherin in Fra-2 TG mice

**FIGURE 4:**
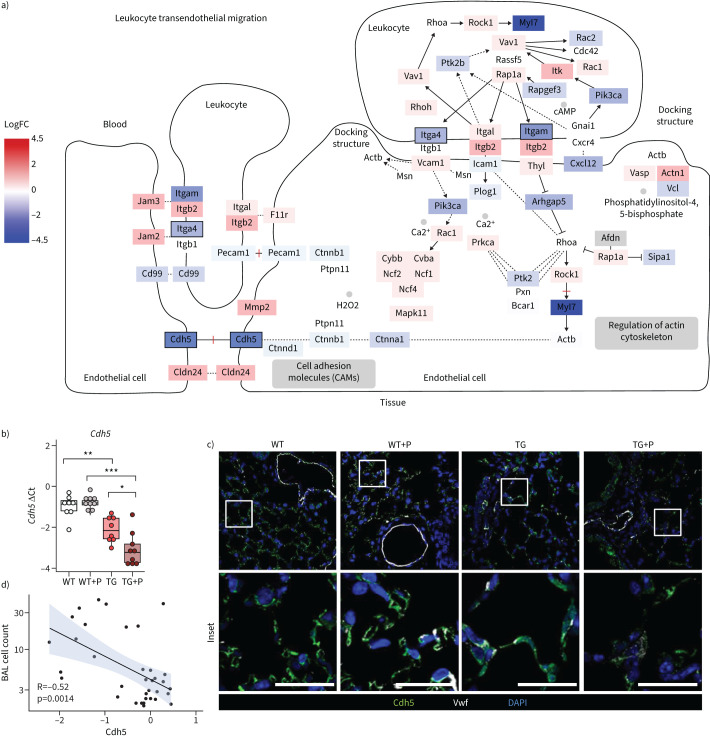
Pirfenidone leads to decreased vascular endothelial (VE)-cadherin expression in the lungs of Fra-2-overexpressing transgenic (Fra-2 TG) mice. a) Schematic of the Kyoto Encyclopedia of Genes and Genomes (KEGG) pathway “Leukocyte transendothelial migration”. The log fold change of gene expression is indicated by colour, significance of regulation is indicated by box borders. b) Quantitative real-time PCR analysis of VE-cadherin (*Cdh5*). Data are presented as boxplots with dot-plot overlays. Statistical analysis was performed using nonparametric Kruskal–Wallis testing with post-analysis to compare specific groups. *: p<0.05, **: p<0.01, ***: p<0.001. c) Low- and high-magnification immunofluorescence images of VE-cadherin (Cdh5) and von Willebrand factor (Vwf) staining in lung tissue from wild-type (WT) and Fra-2 TG mice with (+P) and without pirfenidone treatment. Nuclear counterstain was performed using 4′,6-diamidino-2-phenylindole (DAPI). Scale bars=10 µm. d) Spearman's rank correlation analysis of inflammatory cells in the bronchoalveolar lavage fluid (BAL cell count) and Cdh5 expression.

Inflammatory cell recruitment strongly depends on interactions of inflammatory and endothelial cells, enabling adhesion, rolling and transmigration through the endothelial cell layer. As expression of several endothelial cell junction proteins was decreased (figure 3e), we investigated in detail whether the exaggerated inflammatory response induced by pirfenidone in Fra-2 TG mice was due to an effect on the endothelium. In our transcriptomic dataset, expression of many genes within the Kyoto Encyclopedia of Genes and Genomes pathway “Leukocyte transendothelial extravasation” (mmu04670) were altered, including a significant downregulation of vascular endothelial cadherin (VE-cadherin/Cadherin 5 (Cdh5)) (figure 4a). Indeed, we could confirm a downregulation of Cdh5 gene expression in Fra-2 TG, but not WT mice, following pirfenidone treatment (figure 4b). *Cdh5* was already lower in Fra-2 TG compared to WT mice without treatment (figure 4b), pointing towards a disturbed cell barrier in the lungs of this mouse model. Immunofluorescence staining confirmed this observation. WT mice showed a clear VE-cadherin/Cdh5 staining of capillaries with and without pirfenidone treatment (figure 4c), whereas the immunofluorescence signal was diffuse in Fra-2 TG lungs even without pirfenidone. Pirfenidone treatment further decreased signal intensity in Fra-2 TG lungs (figure 4c). This may indicate disturbed endothelial barrier integrity and consequent inflammatory cell infiltration in the lungs of Fra-2 TG mice compared to WT mice, an effect further potentiated by pirfenidone treatment. Indeed, VE-cadherin expression negatively correlated with inflammatory cell counts in the BAL (figure 4d).

### Pirfenidone ameliorates pulmonary remodelling in bleomycin-induced lung fibrosis

**FIGURE 5:**
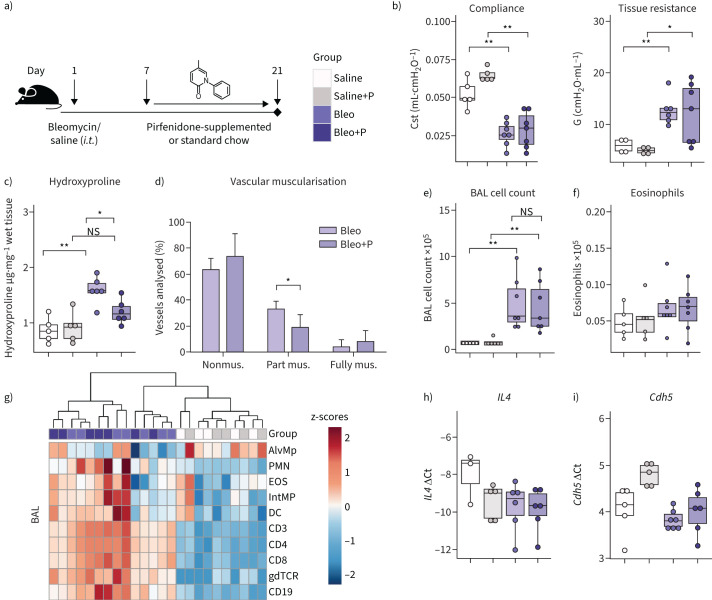
Pirfenidone ameliorates pulmonary remodelling without affecting lung function and inflammation in a bleomycin (Bleo)-induced mouse model of pulmonary fibrosis. a) Schematic representation of pirfenidone (P) treatment in the bleomycin-induced mouse model of pulmonary fibrosis. Lung function measurements and organ collection was performed 21 days after bleomycin and 14 days after pirfenidone treatment. b) Lung function measurements showing quasi-static compliance (Cst) and tissue dampening/resistance (G). c) Hydroxyproline measurement of collagen in lung tissue of saline and bleomycin-treated mice with (+P) and without pirfenidone. Data are presented as boxplots with dot-plot overlays. Statistical analysis was performed using nonparametric Kruskal–Wallis testing with post-analysis to compare specific groups. d) Percentage of nonmuscularised (nonmus.), partially muscularised (part mus.) and fully muscularised (fully mus.) vessels <100 μm in diameter. n=5 (Bleo) or n=7 (Bleo+P). Data are presented as mean±sd. e) Inflammatory cell and f) eosinophil counts in the bronchoalveolar lavage (BAL) of bleomycin and saline-treated mice with (+P) and without pirfenidone treatment. g) Heat map representation with hierarchical clustering of relative proportions of inflammatory cell populations in BAL of bleomycin and saline-treated mice with (+P) and without pirfenidone treatment. Data were normalised using sqrt(sqrt(cellcount)); z-scores are shown. h and i) Quantitative real-time PCR analysis of h) interleukin-4 (*IL4*) and i) vascular endothelial-cadherin (*Cdh5*) gene expression. Data are presented as boxplots with dot-plot overlays. *i.t*.: intratracheal; ns: nonsignificant. *: p<0.05, **: p<0.01.

To investigate whether pirfenidone effects seen in the Fra-2 TG mouse model of SSc-ILD may also be observed in another pulmonary fibrosis model, we investigated the pirfenidone response in a mouse model of bleomycin-induced lung fibrosis. Similar to the experimental design described earlier, control or bleomycin-treated mice received chow supplemented with or without pirfenidone (figure 5a). As expected, bleomycin led to a decline in lung function; however, pirfenidone treatment had no effect on pulmonary compliance and tissue resistance, irrespective of bleomycin or saline pre-treatment (figure 5b). Hydroxyproline analysis revealed a significant decrease in collagen levels in bleomycin-treated mice following pirfenidone administration (figure 5c). We previously reported vascular remodelling with increased muscularisation of small parenchymal vessels in bleomycin-induced lung fibrosis [24]. This vascular remodelling was ameliorated by pirfenidone in bleomycin-treated mice (figure 5d).

Pirfenidone did not alter inflammatory cell counts in the BALF of bleomycin- or saline-treated mice (figure 5e). The number of eosinophils in the BALF of bleomycin-treated mice was unaltered compared to the saline group and not influenced by pirfenidone treatment (figure 5f). Unbiased hierarchical clustering of inflammatory cell populations clearly separated saline and bleomycin groups; however, no clustering was observed according to pirfenidone treatment (figure 5g). Furthermore, both IL-4 and VE-cadherin expression levels were unaffected by pirfenidone treatment in the lungs of bleomycin mice (figure 5h and i). Cumulatively, our data indicate that although lung function was not improved by pirfenidone, application of this drug led to reduced collagen deposition and vascular remodelling in the lungs of bleomycin-treated mice. Importantly, no worsening of pulmonary inflammation was observed.

### Pirfenidone worsens cell barrier function in pre-primed human microvascular endothelial cells

**FIGURE 6:**
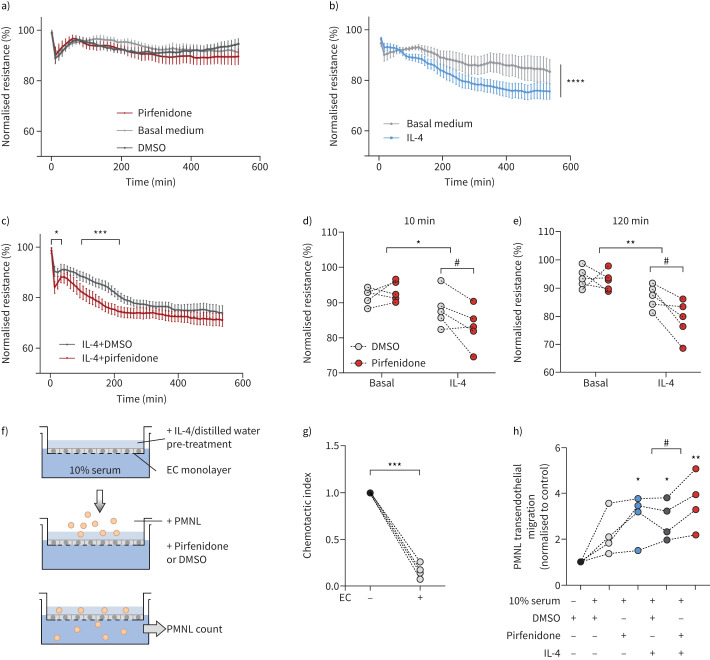
Priming with interleukin (IL)-4 sensitises human lung microvascular endothelial cells (HMVECs) and leads to increased loss of barrier function and increased polymorphonuclear leukocyte (PMNL) transmigration upon pirfenidone treatment. a) Electric Cell-substrate Impedance Sensing (ECIS) measurement of HMVEC barrier resistance in response to pirfenidone. Dimethyl sulfoxide (DMSO) and basal medium served as vehicle and negative control, respectively. b) ECIS measurements of HMVEC barrier resistance in response to IL-4 alone compared to basal medium (vehicle control distilled water). c) ECIS measurements of HMVEC barrier resistance in response to IL-4 in combination with pirfenidone or DMSO vehicle control. d and e) Detailed analysis of b) and c) at d) 10 min and e) 120 min post-treatment. Statistical analysis was performed using two-way ANOVA with multiple comparison testing. IL-4 effect: *: p<0.05, **: p<0.01, ***: p<0.001; pirfenidone effect: ^#^: p<0.05. f) Schematic representation of the transendothelial migration experimental setup. Endothelial cells (ECs) were cultured on 3 µm transmembrane inserts and PMNLs (consisting of eosinophils and neutrophils) were allowed to migrate to 0% or 10% fetal bovine serum, respectively, in the presence or absence of IL4 and/or pirfenidone. g) Transendothelial migration of PMNLs through 3 µm transwell insets with and without HMVEC monolayers in basal medium with 0% serum. h) Transendothelial migration of PMNLs through established HMVEC monolayers in the presence of IL-4 and/or pirfenidone and corresponding vehicle controls. Statistical analysis was performed by one-way ANOVA with Dunnett's post-test using 0% serum with DMSO as control. *: p<0.05, **: p<0.01; t-test was used to compare IL-4 with and without pirfenidone treatment. ^#^: p<0.05.

To elucidate whether increased inflammatory cell recruitment after pirfenidone treatment *in vivo* was linked to downregulation of VE-cadherin and disturbance of endothelial barrier function, we monitored the resistance of human lung microvascular endothelial cells (HMVECs) in response to pirfenidone. Pirfenidone or its vehicle control (dimethyl sulfoxide (DMSO)) alone did not affect HMVEC resistance (figure 6a). As IL-4 is a potent Th2 cytokine leading to increased permeability of endothelial cells [30] and as it was significantly upregulated in Fra-2 TG mice (figure 2f and g), we speculated that priming of endothelial cells through inflammatory factors, such as IL-4, may lead to exaggerated barrier disturbances upon pirfenidone exposure. Pre-treatment of HMVECs with IL-4 led to a steady decrease of barrier function over a time period of 600 min (figure 6b). When challenging IL-4-primed HMVECs with pirfenidone, the loss of endothelial cell resistance was aggravated and observed at earlier time points than after IL-4 alone or IL-4 with vehicle control DMSO (figure 6b and c). A significant drop in resistance was visible after 10 min of combined treatment with IL-4 and pirfenidone, as compared to IL-4 treatment alone or with DMSO vehicle control (figure 6d). Furthermore, the IL-4-induced reduction in barrier resistance 2 h post-treatment was aggravated by pirfenidone (figure 6e).

In order to investigate whether the loss of barrier function induced by pirfenidone in an IL-4-dominated environment could contribute to the increased inflammatory cell recruitment observed in pirfenidone treated Fra-2 TG animals (figure 2), we performed an *in vitro* transendothelial migration assay (figure 6f). The presence of an endothelial monolayer strongly reduced polymorphonucelar leukocyte (PMNL) chemotaxis (figure 6g). Pre-treatment of endothelial cells with IL-4 increased the serum-induced endothelial transmigration of PMNL, which was further enhanced by stimulation with pirfenidone (figure 6h). Of note, treatment of HMVECs with pirfenidone alone induced PMNL transmigration comparable to IL-4 treatment (figure 6h). Taken together, pirfenidone led to loss of endothelial cell barrier function with concomitant increase in leukocyte transmigration in an IL-4-dominated environment *in vitro.* These alterations mimic the observed reduction of VE-cadherin expression and aggravated inflammatory infiltrates in the lungs of Fra-2 TG mice *in vivo*.

## Discussion

In this study, we describe the detrimental effects of pirfenidone on the pulmonary endothelium. Using a mouse model of SSc-ILD, we show how pirfenidone exacerbates pulmonary inflammation, fibrosis and vascular remodelling. Pirfenidone is an approved treatment option for IPF, but not for other non-IPF interstitial lung diseases, such as SSc-ILD. First trials showed that pirfenidone was well tolerated in patients with SSc-ILD [31], yet several recent clinical trials have reported no [32] or weak benefits [16]. Even though the recent RELIEF study did not show serious adverse events in progressive fibrotic interstitial lung diseases other than IPF, the study was terminated prematurely on the basis of an interim analysis for futility triggered by slow recruitment. Subgroup analysis for SSc-ILD could not be performed, as only eight SSc-ILD patients were enrolled [16]. As the role for pirfenidone in SSc-ILD is still unclear, animal models, such as the Fra-2 TG model, present a valuable tool to investigate its potential benefits or drawbacks in SSc-ILD.

Fra-2 TG mice are increasingly used as a model of SSc and SSc-related lung involvement. Fra-2 TG mice not only develop pulmonary fibrosis, but also reflect several important features of SSc-ILD, including vasculopathy with endothelial cell apoptosis and pulmonary hypertension [18, 19], systemic inflammation predominated by Th2 inflammation [17, 20, 21], followed by fibrosis of skin, lung and other organs [17, 22]. Therefore, this model enables pre-clinical investigations and proof-of-concept studies in the context on SSc-ILD. The Fra-2 TG model has already been applied to investigate the efficacy of nintedanib, another antifibrotic drug, recently approved for the treatment of SSc-ILD [23, 33]. In the Fra-2 TG model, nintedanib has been shown to ameliorate pulmonary vascular remodelling as well as parenchymal fibrosis, and additionally prevented endothelial cell apoptosis [23].

Here, we tested the effects of pirfenidone and provide evidence that pirfenidone enhances inflammation, parenchymal and vascular remodelling, as well as worsening lung function in the Fra-2 TG mouse model. In contrast, in the bleomycin-induced lung fibrosis model pirfenidone treatment ameliorated pulmonary remodelling and collagen production, but it did not improve lung function parameters. This agrees with several studies reporting beneficial effects of pirfenidone on collagen deposition; however, there have been inconsistent reports describing its effects on lung function [10, 11]. This strong model-specific response indicates an important interaction of pirfenidone with the underlying model specific pathomechanisms. This is of special interest, as the molecular processes underlying IPF and non-IPF progressive fibrosing lung diseases such as SSc-ILD are diverse and poorly understood [34]. While cellular injury is considered a common trigger for both disease entities, the current paradigm points to injury of epithelial cells in IPF on the one hand, and endothelial cells in SSc-ILD on the other hand [34]. Furthermore, immune alterations in SSc-ILD compared to IPF are distinct. This is highlighted by IL-4 serum levels, which are elevated in SSc-ILD, but not in IPF [35, 36]. In agreement with this, we could show that IL-4 expression levels were elevated in the Fra-2 TG mouse model of SSc-ILD, but not in the bleomycin-induced model of lung fibrosis.

This background information, as well as the fact that negative effects of pirfenidone were only observed in Fra-2 TG, but not in WT mice, triggered the hypothesis that these adverse effects of pirfenidone might be due to the primed/activated vascular endothelium in the inflammatory setting of Fra-2 TG mice.

Pathological changes of the peripheral vascular endothelium are central in SSc from early disease stages onwards, and include loss of adhesion junctions, barrier breakdown, vascular leakage and altered cell extravasation/migration [37, 38]. Although the initial trigger for endothelial injury in SSc remains unknown, inflammatory components could potentiate endothelial activation and dysfunction. Indeed, already in early SSc, immune cells migrate into the lungs and participate in remodelling of the vascular wall [39]. Unravelling the molecular mechanisms underlying vascular microleakage in SSc could therefore be of paramount importance to elucidate interactions with potential future therapeutics such as pirfenidone. Here, the Fra-2 TG mouse model is of particular importance as it closely reflects many aspects of SSc-ILD, including early alterations of the vasculature which precede the onset of fibrosis [24]. Together with other models of pulmonary fibrosis, it might be a valuable tool to examine different aetiologies and pathomechanisms of ILDs and could help to develop more targeted and personalised treatment approaches.

Our transcriptomic analysis revealed that, in Fra-2 TG mice, pirfenidone activated several pathways involved in chemotaxis of granulocytes and lymphocytes. This was on the one hand due to the increased expression of chemoattractants, such as members of the C-C and C-X-C family of chemokines as well as classical cytokines such as IL-4 or IL-6, and on the other hand due to lower expression of genes involved in endothelial barrier formation (*e.g.* integrins or Carcinoembryonic Antigen-Related Cell Adhesion Molecule-1 (CEACAM1). Knockdown of CEACAM1 is known to modulate endothelial barrier leakiness, to increase leukocyte–endothelial interaction [40], and to enhance the development of cardiac fibrosis [41]. These observations support our hypothesis that the inflammatory status of this model primes the pulmonary endothelium for the loss of barrier integrity and increased susceptibility to injury.

Indeed, Fra-2 TG mice exhibit prominent, partially Th2-driven inflammation with high levels of IL-4 [17, 20]. Our findings and those of others demonstrated that IL-4 led to decreased endothelial barrier function [30]. Of note, similar effects were reported for IL-1β [42], another inflammatory cytokine, whose levels are elevated in this mouse model [20]. This suggests that the observed endothelial disturbances in Fra-2 TG mice are most likely not due to one specific mediator, but the entire inflammatory environment. Mechanistically, IL-1β led to reduced endothelial barrier function, due to the suppression of VE-cadherin/Cdh5 expression [42].

In line with this, we showed that Fra-2 TG mice exhibited lower expression of VE-cadherin and increased lung permeability compared to WT mice [43]. This decrease was even stronger in pirfenidone-treated animals. Of note, VE-cadherin levels were unaltered in bleomycin-treated as compared to saline-control mice, and pirfenidone did not affect VE-cadherin expression in bleomycin-treated mice. VE-cadherin is a key component of endothelial adherens junctions and important for barrier integrity and vascular permeability [44]. Loss of VE-cadherin has been described in patients with SSc [45].

In addition, VE-cadherin is downregulated in the lung in response to inflammatory stimuli causing decreased permeability that correlates with poor prognosis [42, 46, 47]. Loss of VE-cadherin was shown in diverse mouse models of fibrosis, underpinning its importance in endothelial cell homeostasis and thus attenuation of fibrosis development [48, 49].

Since vascular and endothelial alterations are a major disease component of SSc, it is vital to understand the mode of action of pirfenidone, in particular its effect on the endothelium. In the carefully balanced interactions between endothelium and immune system, pirfenidone might trigger a vicious cycle of endothelial injury and immune cell recruitment, leading to exacerbated damage of the endothelium. Based on our experimental data, we speculate that Th2-predominant eosinophilic inflammation and endothelial barrier disturbance may be triggers for unfavourable pirfenidone effects in patients. The clinical relevance of our findings is highlighted by case reports describing increased wheezing and cough, eosinophilic pneumonia and increased numbers of lymphocytes and eosinophils after the administration of pirfenidone in IPF patients [50–52]. Therefore, assessment of IL-4 levels, eosinophilia and soluble VE-cadherin as a possible surrogate marker for endothelial barrier dysfunction might be useful tools to distinguish pirfenidone responders from patients at risk of disease exacerbation. However, no data on circulating VE-cadherin and IL-4/eosinophil levels in IPF patients following pirfenidone are available to date and whether they correlate with treatment response needs to be established in further studies.

A limitation of our study is that it is purely based on animal models and lacks clinical data. Nonetheless, it has valuable translational potential, as the Fra-2 TG mouse model closely resembles human SSc-ILD and has been successfully used to assess the efficacy of nintedanib, recently approved for the treatment of SSc-ILD, or IL-13 blocking antibodies [16, 21, 23]. Furthermore, our *in vitro* data confirmed the endothelial barrier loss upon pirfenidone treatment in inflammatory settings seen *in vivo* in Fra-2 TG mice*.* In conclusion, this study shows that the well-established antifibrotic properties of pirfenidone may be overruled by unwanted interactions with pre-injured endothelium in a setting of high Th2 inflammation. Therefore, assessment of Th2 cytokine and eosinophil levels, together with markers of an activated/injured endothelium could be useful to discriminate patients at risk of adverse effects and potential disease exacerbations from patients who may benefit from pirfenidone treatment.

## Supplementary material

10.1183/13993003.02347-2021.Supp1 **Please note:** supplementary material is not edited by the Editorial Office, and is uploaded as it has been supplied by the author.
Supplementary material ERJ-02347-2021.Supplement

## Shareable PDF

10.1183/13993003.02347-2021.Shareable1 This one-page PDF can be shared freely online.
Shareable PDF ERJ-02347-2021.Shareable
