# Supplementary material for: Pirfenidone exacerbates Th2-driven vasculopathy in a mouse model of systemic sclerosis-associated interstitial lung disease
Source: Eur Respir J. 2022 Oct 20;60(4):2102347. doi: 10.1183/13993003.02347-2021 (PMC9582508; doi:10.1183/13993003.02347-2021)
Supplement: Supplementary file 1 [file ERJ-02347-2021.Supplement.pdf]

Pirfenidone exacerbates Th2-driven vasculopathy in a mouse model of SSc-ILD

Birnhuber Anna[1,2], Jandl Katharina[1,3], Biasin Valentina[1,2], Fließer Elisabeth[1], Valzano Francesco[1], Marsh Leigh M[1], Krolczik Christina[4], Olschewski Andrea[1,5], Wilhelm Jochen[6], Toller Wolfgang[5], Heinemann Akos[3], Olschewski Horst[1,7], Wygrecka Malgorzata[4], Kwapiszewska Grazyna[1,2,8].

[1] Ludwig Boltzmann Institute for Lung Vascular Research Graz, Austria.

[2] Otto Loewi Research Center, Division of Physiology, Medical University of Graz, Graz, Austria.

[3] Otto Loewi Research Center, Division of Pharmacology, Medical University of Graz, Graz, Austria.

[4] Center for Infection and Genomics of the Lung, Universities of Giessen and Marburg Lung Center, Giessen, Germany. Member of the German Center for Lung Research.

[5] Department of Anaesthesiology and Intensive Care Medicina, Medical University of Graz, Graz, Austria.

[6] Department of Internal Medicine, Universities of Giessen and Marburg Lung Center, Giessen, Germany.

[7] Division of Pulmonology, Department of Internal Medicine, Medical University of Graz, Graz, Austria.

[8] Institute for Lung Health (ILH), Justus Liebig University, Giessen, Germany

*Corresponding author:

Grazyna Kwapiszewska

Ludwig Boltzmann Institute for Lung Vascular Research

Neue Stiftingtalstrasse 6/VI

8010 Graz, Austria

Phone number: +43 316 385-72918

E-mail: Grazyna.Kwapiszewska@lvr.lbg.ac.at

SUPPLEMENTAL MATERIALS

MATERIAL AND METHODS

Animal experiments

Female Fra-2 overexpressing/transgenic (Fra-2 TG) mice and wild-type (WT) littermates were maintained under specific pathogen free conditions in isolated ventilated cages with 12-hour light/dark cycles. All animal experiments met EU guidelines 2010/63/EU and were approved by the local authorities (Austrian Ministry of Education, Science and Culture). Bleomycin was given intra-tracheally at a dose of 0.8 units per kg bodyweight as described previously [1, 2]. Pirfenidone treatment protocol was adapted from previous publications [3] and incorporated into standard laboratory chow at 2.8 mg/g (Sniff, Soest, Germany). Pirfenidone was provided by HOFFMANN-LA ROCHE. Food was weighed regularly and mean pirfenidone uptake was calculated. Mean pirfenidone uptake was approximately 400 mg/kg bodyweight per day, a frequently used dosage in mouse studies, leading to pirfenidone exposure ratios and plasma concentrations similar to those in patients treated with pirfenidone [4–6]. Pirfenidone treatment of Fra-2 TG and WT mice was performed in two independent experiments with five to eight mice per group. Schematic representations of Fra-2 TG and bleomycin experiments are shown in Figures 1A and 5A, respectively. Treatment of Fra-2 TG and WT mice with the glucocorticoid budesonide was described and published previously [7].

Lung function measurements

Following pirfenidone treatment, lung function of Fra-2 TG and WT littermate control mice with and without pirfenidone treatment was measured. To this end, mice were anesthetized using a combination of 150 mg/kg ketamine and 20 mg/kg xylazine, intubated and mechanically ventilated (frequency: 150 breaths/min, tidal volume: 10 ml/kg and positive end expiratory pressure: 2 cmH2O). Lung function was measured using a flexiVent FX1 module (SciReq, Canada).

Bronchoalveolar lavage fluid (BALF)

Following lung function measurements, mice were sacrificed by exsanguination and lungs were lavaged with 1 ml PBS containing protease inhibitor cocktail (Roche) and 1 mM EDTA. BALF was centrifuged and total cell counts were made, the supernatant was frozen and stored at -80°C until further analysis.

Single cell lung tissue homogenates

Single cell suspensions from lung tissue were performed as previously described [3]. In short, the lower right lobe was digested in medium containing 0.7 mg/ml collagenase and 30 µg/ml DNAse for 40 min at 37°C. Tissue was passed through a 100 µm cell strainer to obtain a single cell suspension. Erythrocytes were lysed for 5 min using ammonium chloride buffer.

Inflammatory cell profiling (Flow cytometry)

BAL cells and single cell lung tissue homogenates were analysed using a LSRII flow cytometer and analysed with the FACSDiva software (BD Biosciences) as previously described [4]. Cells were initially gated on CD45 positivity and were identified as follows: neutrophils (CD11b+, CD11c-, Gr-1+), macrophages (CD11b low, CD11c+, Siglec-F+), dendritic cells (CD11b+, CD11c+, MHC-II high), T helper cells (CD3+, CD4+), cytotoxic T cells (CD3+, CD8+), B cells (CD19+), and eosinophils (CD11b+, CD11c-, Siglec F+). Antibody details are provided in Table S1. For the heatmap presentation, data was normalised using sqrt(sqrt(cellcount)). z-scores cell counts are shown.

Immunofluorescence and tissue staining

Lung tissue samples were formalin-fixed, paraffin embedded and cut into 2.5 µm sections. Sections were deparaffinised and rehydrated in decreasing concentrations of ethanol. Tissue sections were stained with Masson's trichrome or Sirius red for histological collagen analysis. Eosinophils were stained using 1% Chromotrop 2R (St. Louis, MO, US) in aqueous solution for 10 min, followed by hemalaun nuclear counterstain. To quantify the degree of muscularisation, lung tissue sections were stained by double immunohistochemistry with the

endothelial cell marker von Willebrand factor (vWF) and α-smooth muscle actin (α-Sma) as described previously [5].

Immunofluorescence staining was performed on rehydrated tissue sections with antigen retrieval performed in sodium citrate pH6 buffer. Blocking was performed using 5% donkey serum. Sections were incubated with primary antibodies against VE-Cadherin (1:50, R&D Systems) and von Willebrand factor (1:500, Dako) overnight at 4°C, followed by washing and incubation with Alexa Fluor-488/647-labelled secondary antibodies (Life Technologies) at room temperature for 30 min. Negative controls were performed alongside by omission of the first antibody. Slides were mounted with Vectashield DAPI containing mounting medium (Vector Laboratories, Burlingame, CA).

Chamber slides were blocked with 0.1% Triton X and 3% BSA in PBS for one h at room temperature. Unconjugated primary antibodies were diluted in the same buffer and slides were incubated overnight at 4°C. The following day, slides were incubated with Alexa Fluor-488/647-labelled secondary antibodies and AF555 pre-labelled anti-Phalloidin antibody. Slides were mounted with Vectashield DAPI containing mounting medium (Vector Laboratories). All images were taken using a laser scanning confocal microscope (Nikon A1R Ultra-Fast Spectral Scanning Confocal Microscope) with a CFI Plan Apochromat Lambda 60x/1.4 oil immersion objective.

Quantification of vascular remodelling and histological scoring

Tissue sections were analysed using Visiopharm integrated software VIS (Visiopharm, Denmark). Per mouse, 150±67 (minimum: 34, maximum 234) vessels, ranging from 10 to 100 µm in size, were analysed. Deposition of collagen was analysed on Sirius red stained sections, as described previously [5]. To acquire values indicative of parenchymal collagen deposition, bronchi and vessels with a diameter larger than 200 µm were excluded from analysis.

Hydroxyproline measurements

Lung tissue was weighed and hydrolized in 2 N NaOH for eight hours at 120°C. Hydroxyproline measurements were performed as previously published [10].

Western blotting

Proteins from mouse lung homogenate samples were isolated using RIPA buffer (Sigma). Protein samples were separated by SDS-PAGE and transferred to PVDF membranes (GE Healthcare, UK). Membranes were incubated with primary antibodies (Collagen 1, 1:1000; Southern Biotech, USA, and α-tubulin, 1:1000; Cell Signaling Technologies, USA) at 4°C overnight, followed by one h incubation at room temperature with HRP-conjugated secondary antibodies. Membranes were incubated with ECL prime developing solution (GE Healthcare, UK). Signal detection was done using a ChemiDoc Touch Imaging System (Bio-Rad, USA).

RNA isolation and real-time RT-PCR

Total RNA was isolated from lung homogenates using the peqGOLD Total RNA Kit (Peqlab, Germany) and reverse transcribed using the iScript cDNA Synthesis kit (Bio-Rad, USA). The real-time RT-PCR reaction was run on a LightCycler 480 System (Roche Applied Science, Austria) using the QuantiFast SYBR Green PCR kit (Qiagen, Germany). Hydroxymethylbilane synthase (HMBS) and beta-2-microglobulin (B2M) were used as reference genes. The threshold cycle (Ct) difference was calculated as follows: ΔCt = mean Ct reference genes – Ct target gene.

Transcriptomic profiling

Total RNA was isolated from lungs using the RNeasy Mini kit (Peqlab, Erlangen, Germany). 200 ng of total RNA was pre-amplified and labelled with Cy5 using the Low-input QuickAmp Kit (Agilent Technology, Santa Clara, CA) according to the manufacturer's instructions. Hybridizations were performed for 18 h at 42°C on Agilent 6x80K mouse microarrays in Agilent hybridization chambers. Data were analysed using the limma package in R. Intensity values were background-corrected and quantile normalized. Significance of differential expression was estimated using moderated t-statistics as previously described in full [6].

Data visualization was performed in R programming environment using Rstudio (version 1.3.952). Volcano plots were computed using "EnhancedVolcano" Bioconductor package version 1.6.0. Variable genes across the two experimental groups were identified imposing a

significance threshold (-log adjusted p-value) of 1.3 and FC of 1. Colour coding was assigned according to higher enrichment in wild-type animals or transgenic animals. Heatmaps were computed using “pheatmap” package version 1.0.12. Hierarchical clustering was performed to address similarities in each experimental animal in the two groups, respectively. Differential gene expression is shown in single animals and colour coded according “RdYlBl” color palette. Gene Ontology analysis was performed using enrichr [7–9] using the Gene Ontology Biological Process 2018 reference database. P-value adjustment was performed with Benjamini-Hochberg multiple test correction method. Top 10 GO enrichment terms were identified by filtering out terms with adjusted p-value >0.05 and colour coded according to significance power (-log adjusted p-value > 1.3). Chord diagram was computed using “chorddiag” package version 0.1.3 using as input gene ontology results.

Isolation of eosinophils

Blood for the isolation of human eosinophils was collected from healthy volunteers according to a protocol approved by the Ethics Committee of the Medical University of Graz (17-291 ex 05/06), as previously described [10]. In brief, citrated blood was centrifuged at 400 x g, for 20 min to remove plasma. Erythrocytes were eliminated via dextran sedimentation. Peripheral blood mononuclear cells (PBMCs) and polymorphonuclear leukocytes (PMNL) were separated via density gradient centrifugation on Histopaque 1.077 layer. Eosinophil isolation was done from the PMNL fraction by negative selection using the MACS cell separation system, containing a cocktail of biotin-conjugated monoclonal antibodies against CD2, CD14, CD16, CD19, CD56, CD123, and CD235a (Glycophorin A) (Eosinophil Isolation Kit, Miltenyi Biotec, Bergisch Gladbach, Germany), according to manufacturer’s protocol. Purity typically yielded >98 %.

Eosinophil shape change

Eosinophil shape change was measured as previously described [10]. In short, purified eosinophils were kept in assay buffer containing Ca^{2+} and Mg^{2+} ($1x10^6$/mL). Cells were equally divided into reaction tubes and serial dilutions of pirfenidone or eotaxin-2 (diluted in assay

buffer) were added. Cells were incubated in the water bath for 4 min at 37°C followed by immediate transfer to ice and addition of 150 µl of fixative solution on ice, to stop the reaction. Cells were analysed on a FACSCaliburTM (BD Biosciences, NJ, US) and eosinophil shape change was detected as an increase of the forward scatter and was normalized to the respective vehicle control.

Eosinophil chemotaxis

Eosinophil chemotaxis was assessed as previously described [10]. Isolated human eosinophils were resuspended in assay buffer with Ca^{2+} and Mg^{2+} ($1x10^6$/mL), with or without pirfenidone pre-treatment, and placed into top wells of a micro-Boyden chamber with 3 µm pores (NeuroProbe Inc, Gaithersburg, MD, USA) and incubated at 37°C. Eosinophils were allowed to migrate towards increasing concentrations of eotaxin-2 (CCL24, Immunotools, Friesoythe, Germany) for one h. Migrated cells in the bottom wells of the chambers were counted by flow cytometry.

Eosinophil reactive oxygen species production

Production of reactive oxygen species (ROS) was measured as previously described [10]. Isolated human eosinophils were resuspended in assay buffer with Ca^{2+} and Mg^{2+} ($1x10^6$/mL) containing 5 µM dihydrorhodamine 123 and 100 µM pirfenidone, for 30 min at 37°C. Recombinant C5a peptide served as positive control. To stop the reaction samples were transferred to ice, fixed and analyzed by flow cytometry. ROS production was detected as an increase of fluorescence in the FL-1 channel due to the oxidization of the non-fluorescent dye dihydrorhodamine 123 into fluorescent rhodamine 123. Data are shown as fold change to vehicle control.

Lung microvascular endothelial cell culture

Human lung microvascular endothelial cells (HMVEC) purchased from LONZA were cultured in EGM^{TM}-2 endothelial cell media supplemented with microvascular endothelial cell supplementary kit C-22121 (Lonza, Basel, Switzerland) T75 flasks coated with 1% gelatine as previously reported [11]. HMVEC were cultured on 8-well chamber slides. When reaching

confluence, medium was changed to EBM™-2 basal medium supplemented with 2.5% fetal bovine serum and 1% Penicillin/Streptomycin overnight. Next day, cells were pre-treated with IL-4 (20 ng/ml, Immunotools, Friesoythe, Germany), followed by treatment with 500 µM pirfenidone dissolved in DMSO. DMSO alone served as negative control. After 15 min, cells were washed, fixed with 4% PFA in PBS Plus buffer supplemented with 1% BSA and stored at 4°C until further use.

Electrical cell-substrate impedance sensing

Human lung microvascular endothelial cells (LONZA, Basel, CH) were cultured in EGM™-2 endothelial cell growth medium (LONZA, Basel, Switzerland) on 8W10E+ PET Chips pre-activated with 10 mM L-cystein (AppliedBiophysics, NY, USA) until reaching confluence as previously reported [11, 12]. Medium was changed to EBM™-2 basal medium supplemented with 2.5% fetal bovine serum and 1% penicillin/streptomycin overnight. Endothelial barrier integrity was monitored by electrical cell-substrate impedance sensing (ECIS® Z-Theta, AppliedBiophysics, NY, USA) upon stimulation with IL-4 (20 ng/ml) and pirfenidone 500 µM dissolved in DMSO. The same concentrations of DMSO were used as vehicle control.

Transmigration assay

HMVEC were seeded on 96-well Transwell inserts pre-coated with 1% gelatin (3 µm pore size, polycarbonate membrane) (Corning, Lactan, Austria) at a density of 20.000 cells/well in EGM-2 MV growth medium. The following day, HMVECs were pre-treated with vehicle or IL-4 (20 ng/mL) for two h in basal RPMI media (Thermo Fischer, U.S.A). After two h, HMVEC were treated with pirfenidone or vehicle together with the addition of 0.1 x 10^6 polymorphnuclear cells (PMNL) per well to the upper compartment. PMNL were allowed to migrate to the lower compartment supplied with RPMI with 10% FBS or 0% FBS, respectively, for 2.5 h in humidified incubator at 37°C. Subsequently, PMNL were collected from lower compartment and fixed with 100 µl of fixative solution on ice. Migrated cells were counted by flow cytometry FACSCaliburTM (BD Biosciences, NJ, US).

References

1. Biasin V, Crnkovic S, Sahu-Osen A, Birnhuber A, el Agha E, Sinn K, Klepetko W, Olschewski A, Bellusci S, Marsh LM, Kwapiszewska G. PDGFRα and αSMA mark two distinct mesenchymal cell populations involved in parenchymal and vascular remodeling in pulmonary fibrosis. *American Journal of Physiology-Lung Cellular and Molecular Physiology* 2020; 318: L684–L697.

2. Bordag N, Biasin V, Schnoegl D, Valzano F, Jandl K, Nagy BM, Sharma N, Wygrecka M, Kwapiszewska G, Marsh LM. Machine Learning Analysis of the Bleomycin Mouse Model Reveals the Compartmental and Temporal Inflammatory Pulmonary Fingerprint. *iScience* 2020; 23: 101819.

3. Kehrer JP, Margolin SB. Pirfenidone diminishes cyclophosphamide-induced lung fibrosis in mice. *Toxicology Letters* 1997; 90.

4. Schaefer CJ, Ruhrmund DW, Pan L, Seiwert SD, Kossen K. Antifibrotic activities of pirfenidone in animal models. *European Respiratory Review* 2011; 20.

5. Kakugawa T, Mukae H, Hayashi T, Ishii H, Abe K, Fujii T, Oku H, Miyazaki M, Kadota J, Kohno S. Pirfenidone attenuates expression of HSP47 in murine bleomycin-induced pulmonary fibrosis. *European Respiratory Journal* 2004; 24: 57–65.

6. Kehrer JP, Margolin SB. Pirfenidone diminishes cyclophosphamide-induced lung fibrosis in mice. *Toxicology Letters* 1997; 90: 125–132.

7. Gungl A, Biasin V, Wilhelm J, Olschewski A, Kwapiszewska G, Marsh LM. Fra2 Overexpression in Mice Leads to Non-allergic Asthma Development in an IL-13 Dependent Manner. *Frontiers in Immunology* 2018; 9.

8. Nagaraj C, Haitchi HM, Heinemann A, Howarth PH, Olschewski A, Marsh LM. Increased Expression of p22phox Mediates Airway Hyperresponsiveness in an Experimental Model of Asthma. *Antioxidants & Redox Signaling* 2017; 27.

9. Birnhuber A, Crnkovic S, Biasin V, Marsh LM, Odler B, Sahu-Osen A, Stacher-Priehse E, Brcic L, Schneider F, Cikes N, Ghanim B, Klepetko W, Graninger W, Allanore Y, Eferl R, Olschewski A, Olschewski H, Kwapiszewska G. IL-1 receptor blockade skews inflammation towards Th2 in a mouse model of systemic sclerosis. *European Respiratory Journal* 2019; 54.

10. Kesava Reddy G, Enwemeka CS. A simplified method for the analysis of hydroxyproline in biological tissues. *Clinical Biochemistry* 1996; 29: 225–229.

11. Hoffmann J, Wilhelm J, Marsh LM, Ghanim B, Klepetko W, Kovacs G, Olschewski H, Olschewski A, Kwapiszewska G. Distinct Differences in Gene Expression Patterns in Pulmonary Arteries of Patients with Chronic Obstructive Pulmonary Disease and Idiopathic Pulmonary Fibrosis with Pulmonary Hypertension. *American Journal of Respiratory and Critical Care Medicine* 2014; 190.

12. Xie Z, Bailey A, Kuleshov M v., Clarke DJB, Evangelista JE, Jenkins SL, Lachmann A, Wojciechowicz ML, Kropiwnicki E, Jagodnik KM, Jeon M, Ma'ayan A. Gene Set Knowledge Discovery with Enrichr. *Current Protocols* 2021; 1.

13. Kuleshov M v., Jones MR, Rouillard AD, Fernandez NF, Duan Q, Wang Z, Koplev S, Jenkins SL, Jagodnik KM, Lachmann A, McDermott MG, Monteiro CD, Gundersen GW, Ma'ayan A. Enrichr: a comprehensive gene set enrichment analysis web server 2016 update. *Nucleic Acids Research* 2016; 44.

14. Chen EY, Tan CM, Kou Y, Duan Q, Wang Z, Meirelles G, Clark NR, Ma'ayan A. Enrichr: interactive and collaborative HTML5 gene list enrichment analysis tool. *BMC Bioinformatics* 2013; 14.

15. Theiler A, Bärnthaler T, Platzer W, Richtig G, Peinhaupt M, Rittchen S, Kargl J, Ulven T, Marsh LM, Marsche G, Schuligoi R, Sturm EM, Heinemann A. Butyrate ameliorates allergic airway inflammation by limiting eosinophil trafficking and survival. *Journal of Allergy and Clinical Immunology* 2019; 144.

16. Rittchen S, Rohrer K, Platzer W, Knuplez E, Bärnthaler T, Marsh LM, Atallah R, Sinn K, Klepetko W, Sharma N, Nagaraj C, Heinemann A. Prostaglandin D2 strengthens human endothelial barrier by activation of E-type receptor 4. *Biochemical Pharmacology* 2020; 182.

17. Jandl K, Marsh LM, Hoffmann J, Mutgan AC, Baum O, Bloch W, Thekkekara-Puthenparampil H, Kolb D, Sinn K, Klepetko W, Heinemann A, Olschewski A, Olschewski H, Kwapiszewska G. Basement Membrane Remodeling Controls Endothelial Function in Idiopathic Pulmonary Arterial Hypertension. *American Journal of Respiratory Cell and Molecular Biology* 2020; 63.

Tables

Table S1: Details for antibodies used for flow cytometry analysis of inflammatory cell populations in bronchoalveolar lavage and lung tissue of mice.

Antigen	Label	Panel	Company	Clone	Isotype	Dilution Factor
CD19	BB515	1	BD Bioscience	1D3	Rat IgG2a, κ	1:50
CD4	APC	1	Biolegend	GK1.5	Rat IgG2b, κ	1:100
CD8	PE	1	Biolegend	53-6.7	Rat IgG2a, κ	1:200
CD11b	BV510	2	BD Bioscience	M1/70	Rat IgG2b, κ	1:50
CD11c	ef450	2	eBioscience	N418	Armenian Hamster IgG	1:50
CD3	AF700	1	Biolegend	500A2	Syrian Hamster IgG	1:50
CD24	PerCP Cy5.5	2	BD Bioscience	M1/69	Rat IgG2b, κ	1:500
CD25	APC-Cy7	1	Biolegend	PC61	Rat IgG1, λ	1:50
CD45	PerCP-Cy5.5	1	eBioscience	30-F11	Rat IgG2b, κ	1:200
CD45	AF488	2	eBioscience	30-F11	Rat IgG2b, κ	1:200
CD64	AF647	2	BD Bioscience	X54-5/7.1	Mouse IgG1, κ	1:20
gdTCR	BV421	1	Biolegend	GL3	Hamster IgG	1:50
Gr-1	PE-Cy7	2	Biolegend	RB6-8C5	Rat IgG2b, κ	1:800
MHC-II	APC-Cy7	2	Biolegend	M5/114.15.2	Rat IgG2b, κ	1:400
Siglec F	PE	2	BD Bioscience	E50-2440	Rat IgG2a, κ	1:20

Figure S1: Masson's trichrome stainings of the left lung lobes of WT and TG mice with (+P) and without pirfenidone treatment. Four representative lungs per groups are shown. Scalebar = 1 mm

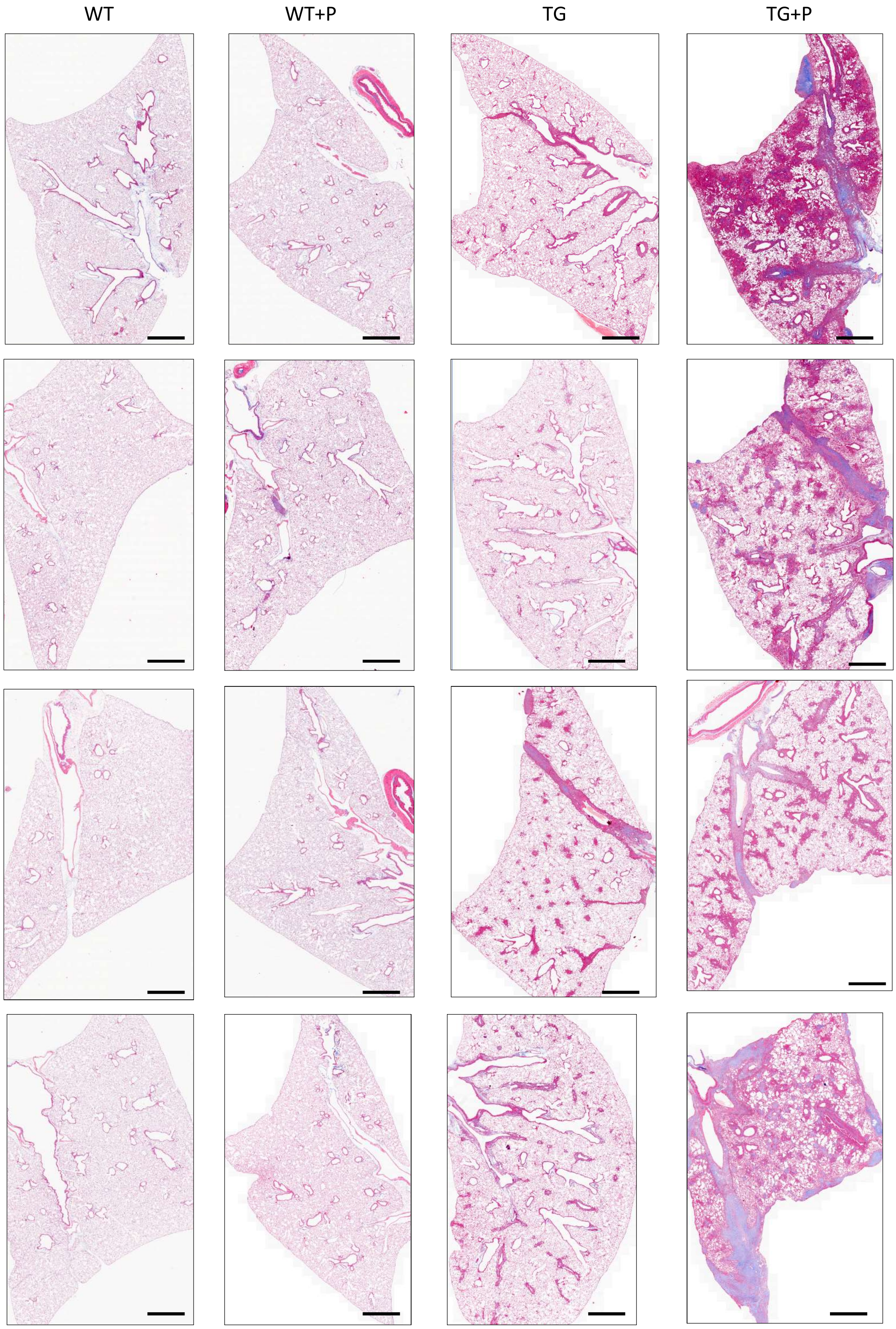
WT
WT+P
TG
TG+P

Figure S2: Budesonide ameliorates parenchymal collagen deposition in Fra-2 TG mice. Quantification of sirius red collagen staining on lung sections from WT and TG mice with (+Bude) and without treatment with the glucocorticosteroid budesonide. Left graph depicts % of collagen within the tissue. Right graph depicts tissue density (% of tissue compared to the whole area analysed).

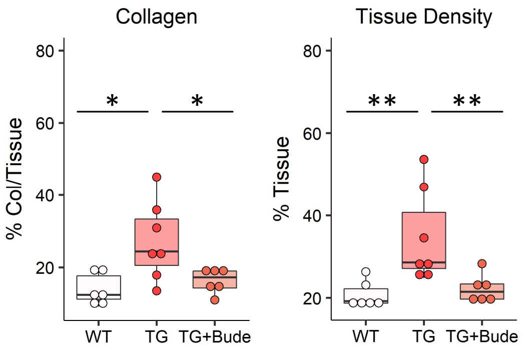

Collagen
% Col/Tissue
80
60
40
20
*
*
WT
TG
TG+Bude
Tissue Density
% Tissue
80
60
40
20
**
**
WT
TG
TG+Bude

Figure S3: Pirfenidone does not exert any direct effect on eosinophil activation, chemotaxis or survival. (A) Shape change measurement of eosinophils in response to pirfenidone or eotaxin-2 with and without pirfenidone pre-treatment. Data are normalised to the corresponding vehicle control. FSC: forward scatter. (B) Chemotaxis measurement (micro Boyden chamber) in response to eotaxin-2 with ant without pirfenidone pre-treatment. (C) Eosinophil ROS production measured by dihydrorhodamine 123 oxidation in response to C5a (positive control) or pirfenidone. Data are shown as fold change to the corresponding vehicle control. (D) Eosinophil survival after 3, 18 or 24 h in medium containing interleukin-5 (IL-5) and/or pirfenidone.

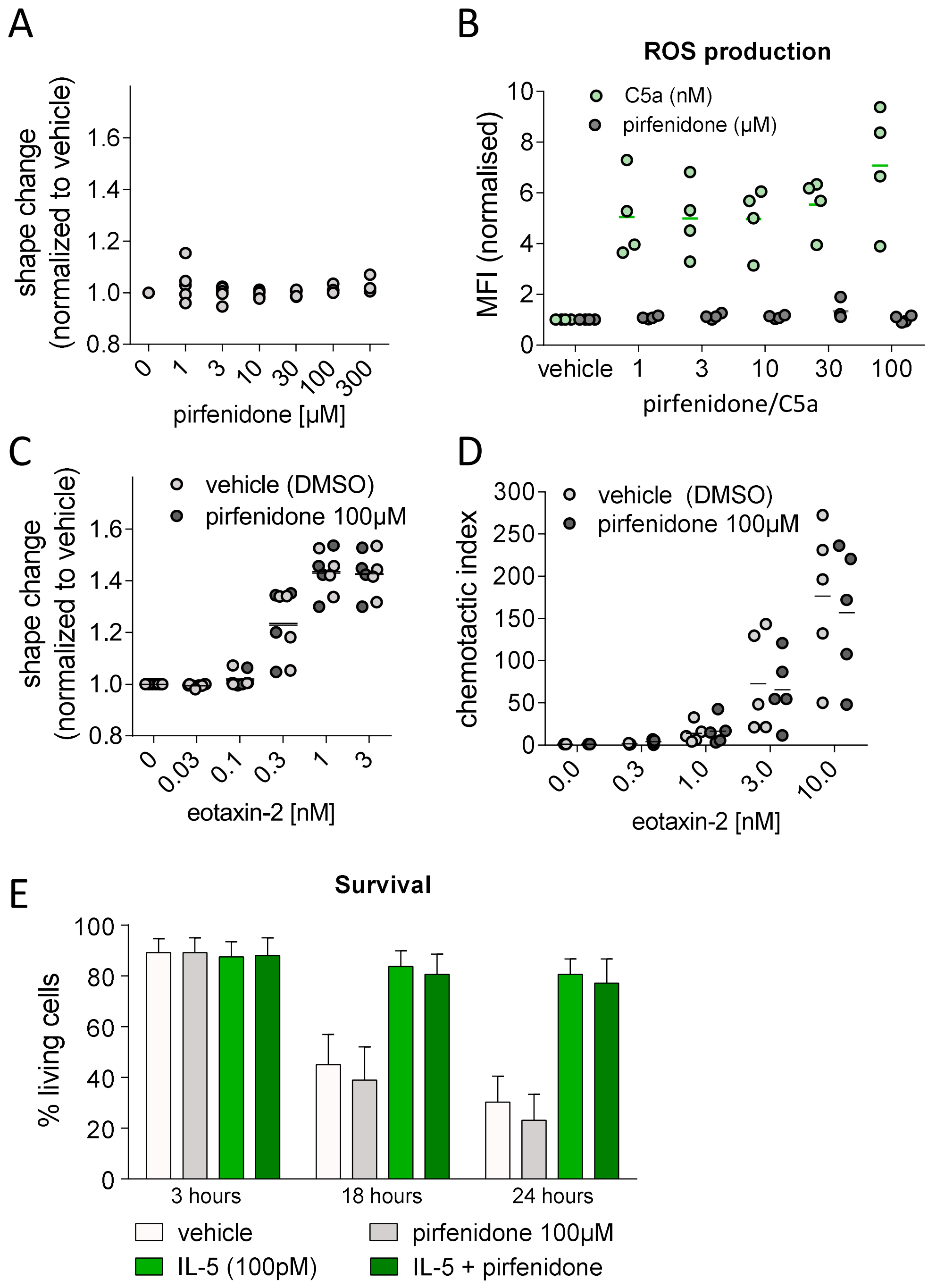
A
shape change (normalized to vehicle)
pirfenidone [µM]
B
ROS production
C5a (nM)
pirfenidone (µM)
MFI (normalised)
vehicle
pirfenidone/C5a
C
vehicle (DMSO)
pirfenidone 100µM
shape change (normalized to vehicle)
eotaxin-2 [nM]
D
vehicle (DMSO)
pirfenidone 100µM
chemotactic index
eotaxin-2 [nM]
E
Survival
% living cells
3 hours
18 hours
24 hours
vehicle
pirfenidone 100µM
IL-5 (100pM)
IL-5 + pirfenidone
