# Supplementary material for: Pirfenidone exacerbates Th2-driven vasculopathy in a mouse model of systemic sclerosis-associated interstitial lung disease
Source: Eur Respir J. 2022 Oct 20;60(4):2102347. doi: 10.1183/13993003.02347-2021 (PMC9582508; doi:10.1183/13993003.02347-2021)
Supplement: Supplementary file 2 [file ERJ-02347-2021.Shareable.pdf]

EUROPEAN RESPIRATORY JOURNAL
ORIGINAL RESEARCH ARTICLE
A. BIRNHUBER ET AL.

SHAREABLE PDF

Pirfenidone exacerbates Th2-driven vasculopathy in a mouse model of systemic sclerosis-associated interstitial lung disease

Anna Birnhuber[1,2], Katharina Jandl[1,3], Valentina Biasin[1,2], Elisabeth Fließer[1], Francesco Valzano[1], Leigh M. Marsh[1], Christina Krolczik[4], Andrea Olschewski[1,5], Jochen Wilhelm[6], Wolfgang Toller[5], Akos Heinemann[3], Horst Olschewski[1,7], Malgorzata Wygrecka[4] and Grazyna Kwapiszewska[1,2,8]

[1]Ludwig Boltzmann Institute for Lung Vascular Research Graz, Graz, Austria. [2]Otto Loewi Research Center, Division of Physiology, Medical University of Graz, Graz, Austria. [3]Otto Loewi Research Center, Division of Pharmacology, Medical University of Graz, Graz, Austria. [4]Center for Infection and Genomics of the Lung, Universities of Giessen and Marburg Lung Center, Member of the German Center for Lung Research, Giessen, Germany. [5]Dept of Anaesthesiology and Intensive Care Medicine, Medical University of Graz, Graz, Austria. [6]Dept of Internal Medicine, Universities of Giessen and Marburg Lung Center, Giessen, Germany. [7]Division of Pulmonology, Dept of Internal Medicine, Medical University of Graz, Graz, Austria. [8]Institute for Lung Health (ILH), Justus Liebig University, Giessen, Germany.

Corresponding author: Grazyna Kwapiszewska (Grazyna.Kwapiszewska@lvr.lbg.ac.at)

Check for updates

Shareable abstract (@ERSpublications)
Pirfenidone worsens vascular and pulmonary dysfunction in a SSc-ILD mouse model due to its negative effects on primed endothelial cells and may lead to unfavourable effects in patients with underlying type 2 inflammation as seen in SSc-ILD https://bit.ly/3Jk821j

Cite this article as: Birnhuber A, Jandl K, Biasin V, *et al*. Pirfenidone exacerbates Th2-driven vasculopathy in a mouse model of systemic sclerosis-associated interstitial lung disease. *Eur Respir J* 2022; 60: 2102347 [DOI: 10.1183/13993003.02347-2021].

This single-page version can be shared freely online.

Copyright ©The authors 2022.

This version is distributed under the terms of the Creative Commons Attribution Licence 4.0.

This article has an editorial commentary: https://doi.org/10.1183/13993003.01301-2022

Received: 27 Aug 2021
Accepted: 8 March 2022

Abstract

Background Systemic sclerosis (SSc) is an autoimmune disease characterised by severe vasculopathy and fibrosis of various organs including the lung. Targeted treatment options for SSc-associated interstitial lung disease (SSc-ILD) are scarce. We assessed the effects of pirfenidone in a mouse model of SSc-ILD.

Methods Pulmonary function, inflammation and collagen deposition in response to pirfenidone were assessed in Fra-2-overexpressing transgenic (Fra-2 TG) and bleomycin-treated mice. In Fra-2 TG mice, lung transcriptome was analysed after pirfenidone treatment. *In vitro*, pirfenidone effects on human eosinophil and endothelial cell function were analysed using flow cytometry-based assays and electric cell-substrate impedance measurements, respectively.

Results Pirfenidone treatment attenuated pulmonary remodelling in the bleomycin model, but aggravated pulmonary inflammation, fibrosis and vascular remodelling in Fra-2 TG mice. Pirfenidone increased interleukin (IL)-4 levels and eosinophil numbers in lung tissue of Fra-2 TG mice without directly affecting eosinophil activation and migration *in vitro*. A pronounced immune response with high levels of cytokines/chemokines and disturbed endothelial integrity with low vascular endothelial (VE)-cadherin levels was observed in pirfenidone-treated Fra-2 TG mice. In contrast, eosinophil and VE-cadherin levels were unchanged in bleomycin-treated mice and not influenced by pirfenidone. *In vitro*, pirfenidone exacerbated the IL-4 induced reduction of endothelial barrier resistance, leading to higher leukocyte transmigration.

Conclusion This study shows that antifibrotic properties of pirfenidone may be overruled by unwanted interactions with pre-injured endothelium in a setting of high T-helper type 2 inflammation in a model of SSc-ILD. Careful ILD patient phenotyping may be required to exploit benefits of pirfenidone while avoiding therapy failure and additional lung damage in some patients.
